# Effects of chronic forced circadian desynchronization on body weight and metabolism in male mice

**DOI:** 10.14814/phy2.12743

**Published:** 2016-04-28

**Authors:** Leandro P. Casiraghi, Ana Alzamendi, Andrés Giovambattista, Juan J. Chiesa, Diego A. Golombek

**Affiliations:** ^1^ Departamento de Ciencia y TecnologíaUniversidad Nacional de Quilmes ‐ CONICET. BernalBuenos AiresArgentina; ^2^ Unidad de NeuroendocrinologíaIMBICE (CONICET‐CICPBA)La PlataArgentina

**Keywords:** Chronic jet‐lag, circadian disruption, restricted feeding, shift work, wheel‐running

## Abstract

Metabolic functions are synchronized by the circadian clock setting daily patterns of food intake, nutrient delivery, and behavioral activity. Here, we study the impact of chronic jet‐lag (CJL) on metabolism, and test manipulations aimed to overcome potential alterations. We recorded weight gain in C57Bl/6 mice under chronic 6 h advances or delays of the light–dark cycle every 2 days (*ChrA* and *ChrD*, respectively). We have previously reported *ChrA*, but not *ChrD*, to induce forced desynchronization of locomotor activity rhythms in mice (Casiraghi et al. 2012). Body weight was rapidly increased under *ChrA*, with animals tripling the mean weight gain observed in controls by day 10, and doubling it by day 30 (6% vs. 2%, and 15% vs. 7%, respectively). Significant increases in retroperitoneal and epidydimal adipose tissue masses (172% and 61%, respectively), adipocytes size (28%), and circulating triglycerides (39%) were also detected. Daily patterns of food and water intake were abolished under *ChrA*. In contrast, *ChrD* had no effect on body weight. Wheel‐running, housing of animals in groups, and restriction of food availability to hours of darkness prevented abnormal increase in body weight under *ChrA*. Our findings suggest that the observed alterations under *ChrA* may arise either from a direct effect of circadian disruption on metabolism, from desynchronization between feeding and metabolic rhythms, or both. Direction of shifts, timing of feeding episodes, and other reinforcing signals deeply affect the outcome of metabolic function under CJL. Such features should be taken into account in further studies of shift working schedules in humans.

## Introduction

Circadian rhythms are ubiquitous in nature and are present at all levels of biological organization, regulating from cell cycle to sleep architecture, including multiple physiological and behavioral variables (Dibner et al. 2010; Golombek and Rosenstein 2010). Metabolic variables, as expected, are also controlled by the circadian system in mammals, including humans (Bailey et al. 2014).

The array of central and peripheral circadian clocks keeps a tight control of energy metabolism and nutrients intake. While feeding provides the organism with energy sources essential to homeostasis, intake does not occur indistinctly across the day, but periodically based on the species natural temporal niche (diurnal or nocturnal). In general, major intake is coincident with wakefulness, locomotion, and high anabolic activity. Inversely, fasting correlates with rest and sleep, and mainly catabolic activity (Challet 2013).

Homeostasis of food intake and energy metabolism in mammals is regulated by the central nervous system through a network of nuclei in the basal hypothalamus and the brain stem. In turn, these structures receive signals from peripheral tissues, as metabolites (glucose, fatty acids) and hormones (ghrelin from the stomach, leptin from adipose tissues, and insulin from the pancreas) (Bailey et al. 2014). Daily and circadian rhythms of food intake depend at least in part on the master circadian clock activity in the suprachiasmatic nuclei (SCN), since it is been long known that lesions in this region abolish feeding rhythms (van den Pol and Powley 1979) and alter metabolic variables such as the response to insulin and gluconeogenesis (Rudic et al. 2004). Moreover, mutations in the so‐called *clock genes* can also affect the patterns of food intake (Loudon et al. 2007). Oscillations in energy expenditure and in the respiratory quotient have been well characterized (Challet 2013), and SCN lesions abolish these variations (Nagai et al. 1985). However, there are experimental evidences that point for a hierarchical SCN control of an integrated sleep‐fasting‐catabolism/wake‐feeding‐anabolism rhythm, involving secondary circadian oscillators in the brain and the digestive tract (Challet 2013).

Circadian interactions between feeding and metabolism are of importance to set energy homeostasis. When animals are fasted, rhythms in circulating metabolites are maintained, demonstrating that these do not depend exclusively upon patterns of intake (Escobar et al. 1998); nevertheless, scheduled food restriction can modify these metabolic rhythms phases (Satoh et al. 2006). Availability of food can also have important effects on circadian function when it is restricted on a daily basis. Indeed, a vast amount of literature indicates the presence of a food‐entrainable oscillator (FEO) regulating rhythmic anticipatory behavior to the presentation of food (Mistlberger 2011), although both the circadian nature and precise localization of such oscillator are still under study. Other features such as body temperature and corticosterone levels are synchronized by restricted feeding regimes (Mistlberger 2011). Even SCN‐lesioned mice can recover certain circadian rhythmicity when fed on a periodical schedule (Mendoza 2007).

Taking this bidirectional interaction between the circadian system and metabolism into account, it should not be surprising that disruption of biological rhythms impose negative effects on metabolism, leading to pathologies at various levels (Turek et al. 2005; Karatsoreos et al. 2011; Barclay et al. 2012). In the same way, metabolic disorders can alter circadian function with equally damaging consequences (Challet 2013). Human night and shift‐workers are known to suffer from a diverse array of health complications (including metabolic syndrome), and recently the World Health Organization has declared shift‐working as “potentially carcinogenic for human beings” (Straif et al. 2007; Golombek et al. 2013; Wang et al. 2014).

The study of animal models of circadian disruption is a key tool to understand the basis of circadian stress‐related disease and develop new alternatives. In this paper, we study the metabolic features of a model of forced desynchronization of the circadian system in mice through chronic jet‐lag (a paradigm for shift working), that we recently developed (Casiraghi et al. 2012), and test potential strategies to prevent the complications observed in the model.

## Methods

### Animals

C57Bl/6 male mice aged 3–4 months from the animal breeding facilities at the National University in La Plata (La Plata, Argentina) were used in all experiments. Upon arrival, animals were housed under a 12 h:12 h light:dark schedule (LD) with water and standard rodent diet ad libitum for at least 2 weeks before entering experimental conditions.

The experimental protocols in this study were prepared according to the ARRIVE guidelines (Animal Research: Reporting In Vivo Experiments), and approved by the Universidad Nacional de Quilmes IACUC (Institutional Animal Care and Use Committee) according to the NIH Guidelines.

### Experimental lighting schedules

**Figure 1:**
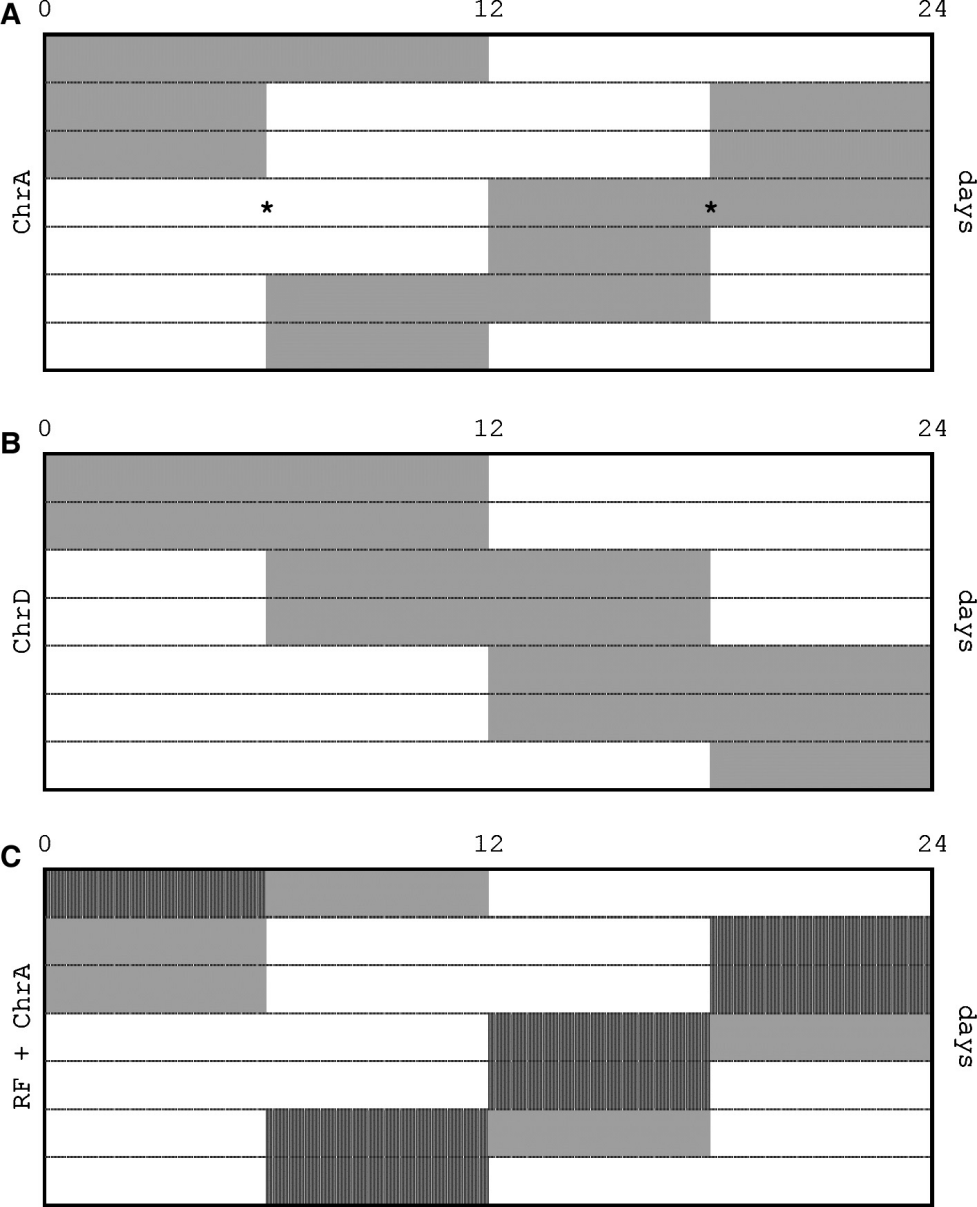
Schematic design of the experimental lightning schedules: each row represents an entire day from 0 to 24 h. Darkness and light periods are indicates by the gray and clear areas, respectively. (A) ChrA consists in the chronic 6 h shortening of every two dark phases. The asterisks exemplify the times across the cycle when blood and tissue samples were obtained. (B) ChrD consists in the chronic 6 h lengthening of every two light phases. (C) RF + ChrA involves the ChrA schedule above with food availability restricted to the first (and in half the cases, only) 6 h of each dark phase (indicated with vertical stripes).

Light intensity in all schedules was set to 200–300 lux at cage level. Besides the control LD schedule already described, two chronic experimental jet‐lag (CJL) schedules were used in the experiments (see [Casiraghi et al. 2012]). For chronic advancing jet‐lag (*ChrA*), mice were subjected to a 6 h advance of the LD cycle every 2 days, accomplished through a 6 h shortening of every second dark phase. For chronic delaying jet‐lag (*ChrD*), mice were subjected to a 6 h delay in the LD cycle every 2 days, through a 6 h lengthening of every second light phase. See Figure 1 for a schematic description of the experimental schedules.

### Weight gain under CJL conditions

All mice were 3–4 months at the start of each experiment, and were housed in individual cages (except when noted) with food and water ad libitum. Body weight and daily food intake was recorded twice a week, first through a “baseline period” under LD conditions for at least 2 weeks and then throughout the whole experimental lighting period, which starts at “day 0”. The weight on day 0 for each animal was used to relativize all previous and subsequent recorded weights, by dividing them to the weight on day 0. This was performed to discard individual differences *intra* groups. Weight gain in groups under each of the CJL conditions tested was compared to groups of littermate animals under control LD conditions. Comparisons performed were: (1) ChrA versus LD (*n* = 17); (2) ChrA versus LD, with animals caged in groups of 3 or 4 (*n* = 22; see notes below); (3) ChrA versus LD, with running wheels available (*n* = 10); and (4) ChrD versus LD (*n* = 10).

In the experiment in (2), as animals were housed in groups, it was not possible to measure food intake in individual animals.

Weight and food intake recording, food and water supply, and bedding and cage changes were scheduled in accordance with the lightning schedules to avoid introducing novel cues from the external time.

### Food restriction to dark phases under ChrA

After 2 weeks in individual cages under baseline conditions (LD, food and water ad‐libitum), on day 0, the animals were divided in four groups (*n* = 6 for each group). Two of these were switched to ChrA, while the remaining groups were kept under the control LD schedule. One of the groups under each lighting condition was subjected to a feeding schedule in which food was made available only during the first 6 h of each dark phase of the light schedule, while water remained freely available (see Fig. 1C). In the remaining groups, individuals were fed ad libitum, although food was removed and readded to the cages along with that of the restricted groups as a sham intervention. In brief, the experiment involved the following groups: (1) mice under ChrA with food ad libitum (AL + ChrA); (2) mice under ChrA with food restricted to the dark hours (RF + ChrA); (3) mice under LD with food ad libitum (AL + LD); and (4) mice under LD with food restricted to the dark hours (RF + LD). Body weight (relative to the weight on day 0) and food intake were recorded every week.

### Locomotor activity rhythms

To measure the general locomotor activity rhythms, infrared motion detectors were set on top of the cages and calibrated individually for each animal. Running wheel locomotor activity was measured using magnetic switches attached to the wheels. Both detectors were connected to a computer interface that records activity counts every five minutes for posterior time‐series analysis (Archron, Buenos Aires, Argentina).

Locomotor activity rhythms were determined for all experimental CJL schedules tested. The effect of running wheel activity, and of the restriction of food availability to darkness on locomotor rhythm synchronization under ChrA, were studied for the first time for this article.

### Determination of metabolic variables

Mice were divided in two groups under normal LD (*n* = 16) or ChrA (*n* = 16) conditions; each group was then subdivided in groups of eight. The animals were weighted and killed by decapitation (JL30 and JL60). Samples were taken either at mid‐light or mid‐dark phases (see Fig. 1A).

From all animals, we obtained: whole blood, whole retroperitoneal and epidydimal adipose tissues, and whole liver samples. Blood was collected in anti‐coagulated tubes, while tissues were removed through dissection and stored in sterile containers by separately.

Triglycerides, leptin, and glucose levels were measured in whole blood. Circulating triglycerides were determined using the TG Colour enzymatic assay (Wiener Lab, Buenos Aires, Argentina; sensitivity = 0.008 g/L, coefficient of variability = 1.82–2.11%) that involves a chromogen later detected by absorbance. Leptin was measured using a validated specific RIA assay (sensitivity = 0.05 ng/mL, coefficient of variability = 5–8%; [Giovambattista et al. 2000]). A commercial assay kit was used to measure plasma glucose levels (Bio System Lab., Buenos Aires, Argentina; sensitivity = 0.23 mg/dL).

After being weighed, retroperitoneal adipose tissue was immediately fixed in 10% formaldehyde for 36 h. After fixation and dehydration, tissue samples were embedded in paraffin for histological observation, which was performed on 4–5 *μ*m slices after hematoxylin–eosin staining. Morphometric and quantitative analyses were performed through a CCD camera coupled to the Image Pro Plus 6.0 software (Mediacy, Rockville, MD). For each sample, seven sections and three levels were chosen (4–5 animals for each group). Fifteen random microscope fields were analyzed for each section, giving a mean of 2500 total adipocytes for which the area was determined in each group through software analysis.

Additionally, around JL30, we measured mean food and water intake during light and darkness phases across three nonconsecutive complete cycles in both the experimental ChrA and the LD control groups (*n* = 8 for each group).

### Statistical analysis

Differences in relative weight gain curves in each experiment were analyzed through two‐way analysis of variance (ANOVA), taking lighting schedules and repeated measures as factors. Differences in specific temporal points were determined using a posteriori Bonferroni contrasts. In the evaluation of restricted feeding schedules, time cuts were analyzed individually using two‐way ANOVA and a posteriori Dunnett contrasts using the AL + LD group as control.

For the comparison of metabolic variables, two‐way ANOVA was used to analyze either differences between light and dark phases or between values at JL30 and JL60, in mice under ChrA or controls. Bonferroni contrasts were used to analyze intragroup differences in each case; that is light versus darkness or JL30 versus JL60.

All the variables analyzed were studied to verify the normal distribution (Chi square test) and homogeneity of variances (Levene test) in data subsets. Hypothesis test was constructed by setting a 95% confidence level for rejecting the null hypothesis by chance (i.e., *P* = 0.05).

ANOVA and contrasts were performed using Graph Pad Prism software (La Jolla, CA). Actograms and periodograms for the analysis of activity rhythms were performed using El Temps software (University of Barcelona, Spain).

All plots presented show data as mean ± standard deviation.

## Results

**Figure 2:**
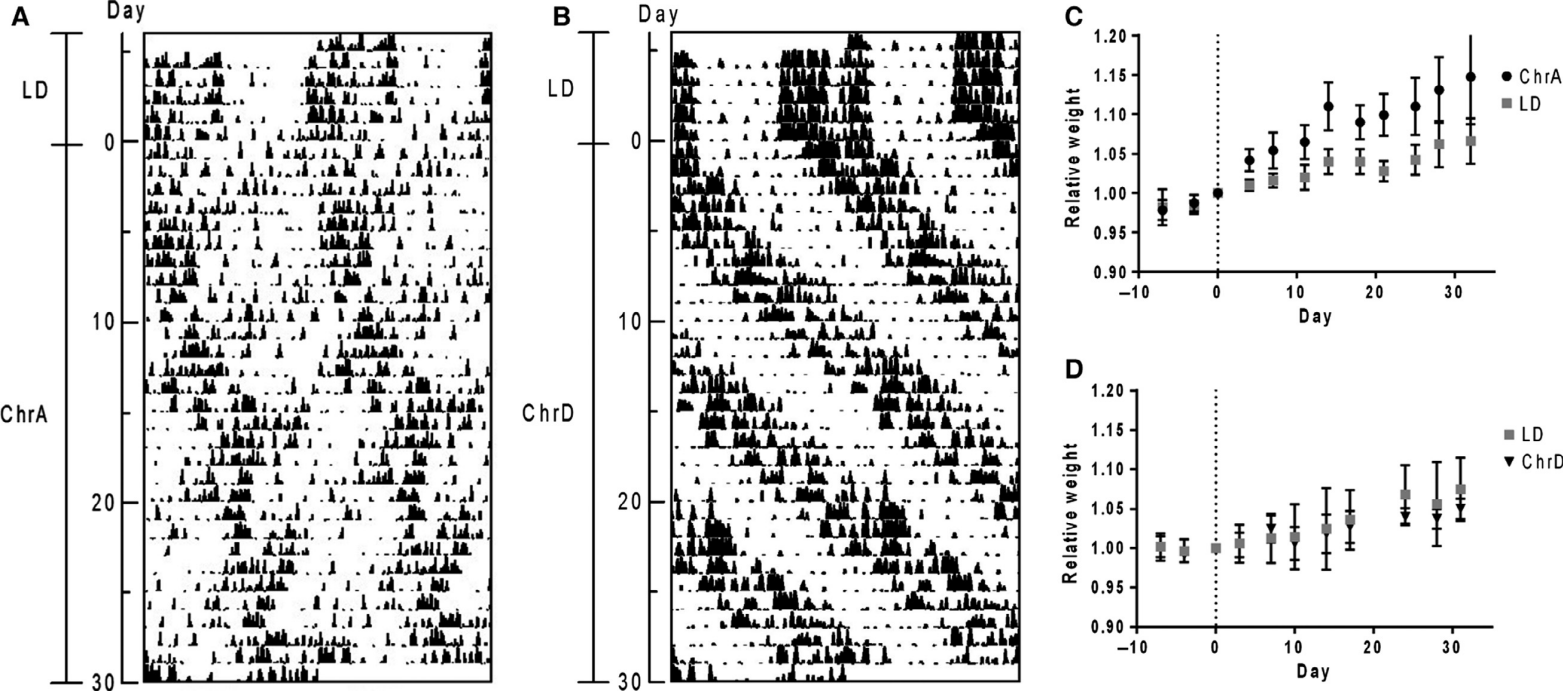
Double‐plot actograms of: (A) locomotor activity of a mouse under the ChrA protocol showing two desynchronized components of activity rhythms; and (B) locomotor activity of a mouse under the ChrD protocol showing correct synchronization to the schedule. The intervals of different light schedules and the days under the experimental schedule are indicated to the left. (C, D) Relative body weight curves across over 30 days of animals: (C) under the ChrA schedule (*n* = 12 and 5 for the ChrA and the LD groups, respectively); and (D) under ChrD (*n* = 5 for each group). Day 0 indicates the day of the start of the CJL schedules, and recorded weights across the experiment are all relativized to the weight on this day for each animal. Curves were analyzed through two‐way ANOVA including repeated measures as a factor. Results: (C) interaction ns., time *P* < 0,0001, jet‐lag *P* < 0,01; (D) interaction ns., time *P* < 0,0001, jet‐lag ns.

We have shown in a previous work that mice under the ChrA protocol display forced desynchronization of general locomotor activity rhythms (Fig. 2A; see [Casiraghi et al. 2012]). In short, forced desynchronized mice under this protocol display two components of locomotor activity, a short‐period one (around 21.0 h) phase locked to the ChrA schedule, and a long period one (around 24.7 h) which runs in relative coordination. We also proposed a mathematical model of the dynamics of the circadian system under CJL schedules that supports the patterns of activity described (Casiraghi et al. 2012).

Under this desynchronization protocol, mice show significantly increased body weight gain rates as compared to those of animals under control LD conditions (Fig. 2C; repeated measures two‐way ANOVA: interaction ns., time under ChrA *P* < 0.0001, jet‐lag *P* < 0.05). Animals under ChrA showed a mean 6% weight increase over the first 10 days and 15% by day 30 (vs. 2% and 7% in the LD group, respectively; see absolute weight values in Table 1). The effect remained consistent after over 60 and 80 days, with animals under ChrA showing a mean 25% and 28% increase in weight, respectively, versus the 15% and 16% increases observed for the control group (mean weight in grams ± SD: 60 days: ChrA = 34.5 ± 3.3, LD = 31.3 ± 2.6; 80 days: ChrA = 35.4 ± 3.7, LD = 31.6 ± 3.3). This effect was independent from total food intake, as both groups consumed similar daily amounts of food across the experiment (mean daily intake in grams ± SD: ChrA = 3.3 ± 0.3, LD = 3.5 ± 0.3; repeated measures two‐way ANOVA: ns. for interaction, jet‐lag and day). In contrast, animals under the delay protocol ChrD, which does not induce desynchronization of locomotor rhythms (Fig. 2B; locomotor activity shows an entrained rhythm with a period of 27 h, also characterized in [Casiraghi et al. 2012]), did not show differences in weight gain nor in food intake (mean daily intake in grams ± SD: ChrD = 3.7 ± 0.6, LD = 3.6 ± 0.4; repeated measures two‐way ANOVA: ns. for interaction, jet‐lag and day) when compared to littermate control mice under LD (Fig. 2D, and Table 1).

**Table 1:** Summary of body weight evolution under different CJL protocols, indicating the *n*, and mean weights (±standard deviation) of each group at days 0, 10 and 30 (±1 day). The *P* column indicates whether differences between groups were significant according to the statistical analysis used (*Chronic advances*,* Chronic delays*,* Advances plus wheel*, and *Advances in groups* experiments: repeated measures two‐way ANOVA; *Restricted feeding* experiment: two‐way ANOVA followed by Dunnett contrasts against the *AL − LD* group, at day 30 [see Fig. 5])

Experiment	Group	*n*	Mean weight ± SD (g)			*P*
			day 0	day ~10	day ~30	
Chronic advances	ChrA	12	27.7 ± 1.5	29.5 ± 1.5	31.8 ± 2.6	<0.01
	LD	5	27.2 ± 1.8	27.8 ± 2.0	29.1 ± 2.6	
Chronic delays	ChrD	5	27.6 ± 1.4	27.8 ± 0.9	29.0 ± 1.8	ns.
	LD	5	26.6 ± 1.2	27.0 ± 1.0	28.6 ± 0.8	
Advances plus wheel	ChrA + W	5	27.5 ± 1.0	27.8 ± 1.9	28.3 ± 2.2	ns.
	LD + W	5	28.8 ± 1.2	27.9 ± 1.1	29.3 ± 1.0	
Advances in groups	ChrA + G	11^a^	25.5 ± 2.0	26.9 ± 2.1	28.9 ± 2.2	ns.
	LD + G	11^a^	25.3 ± 1.2	26.7 ± 1.6	27.8 ± 1.6	
Restricted feeding	AL + ChrA	6	25.8 ± 2.2	27.7 ± 3	28.5 ± 2.9	<0.001
	RF + ChrA	6	27.0 ± 1.1	27.3 ± 0.8	28.1 ± 1.2	ns.
	AL + LD	6	25.8 ± 1.6	26.1 ± 1.6	26.4 ± 1.0	
	RF + LD	6	25.1 ± 1.8	23.9 ± 1.6	25.6 ± 1.7	ns.

a The 11 animals in each experimental group were housed in groups of 3 or 4 mice.

**Figure 3:**
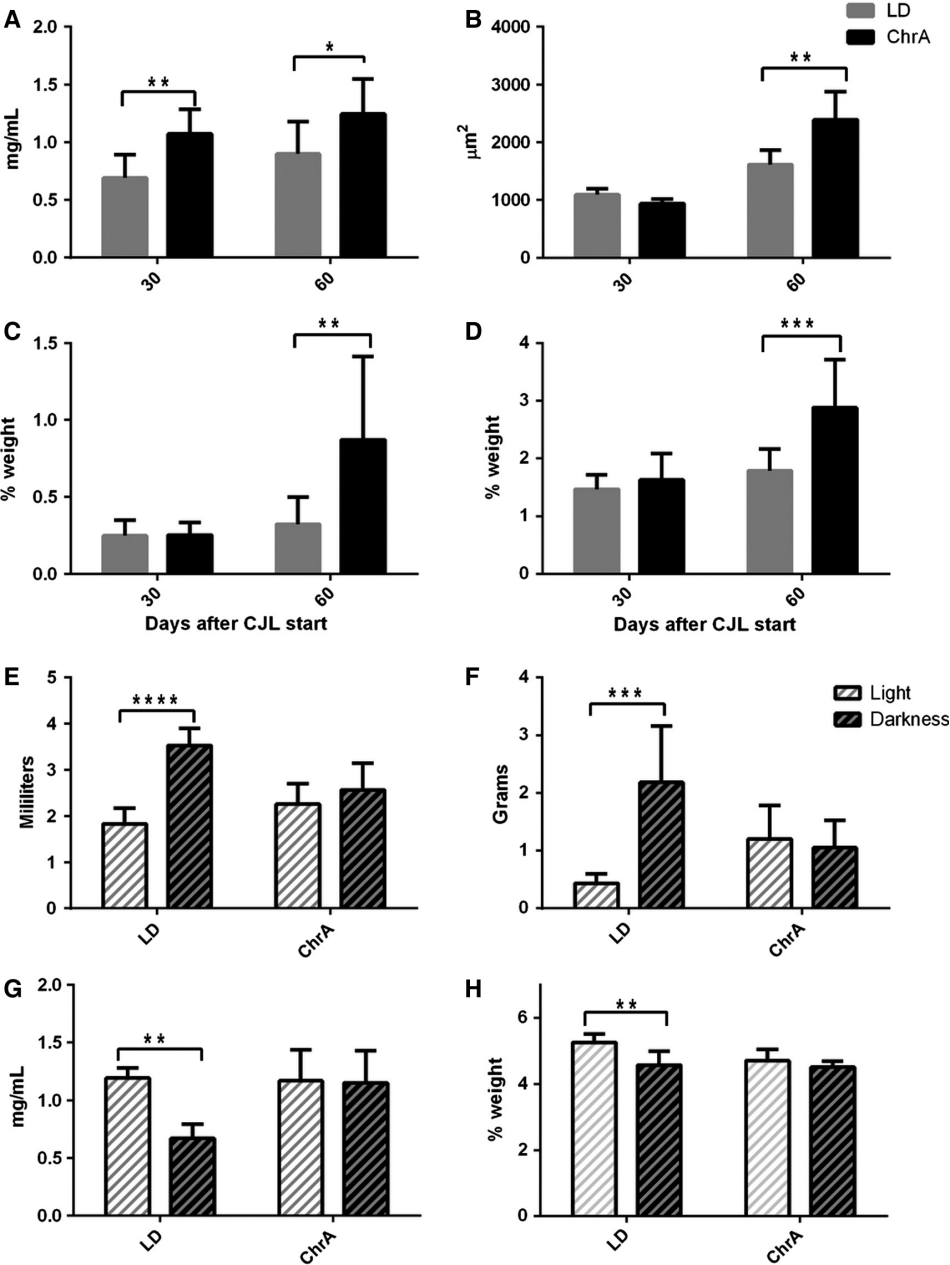
Metabolic variables after 30 and/or 60 days under the ChrA protocol. (A) Triglycerides levels in whole blood at JL30 and JL60 (*n* = 8 for each bar). (B) Mean retroperitoneal adipocytes area (*n* = 4 for each bar). (C) Retroperitoneal and (D) epidydimal adipose tissues masses (as % of body weight) (*n* = 8 for each bar in C–D). (E, F) Total amounts of (E) water and (F) food consumed in average during light and dark phases at JL30 in control and ChrA groups (*n* = 8 for both groups). (G) Triglycerides levels in whole blood at light and dark phases at JL30. (H) Liver weight (as % of body weight) at light and dark phases at JL30 (*n* = 4 for each bar in g and h). Two‐way ANOVA analysis was performed in each case, with a posteriori Bonferroni contrasts within levels: 30 and 60 days in A–D; and LD and ChrA in E–H (asterisks indicate significant *P* values). Two‐way ANOVA results: (A) interaction ns., day *P* < 0.05, jet‐lag *P* < 0.001; (B) interaction *P* < 0.001, day *P* < 0.0001, jet‐lag *P* < 0.05; (C) interaction *P* < 0.05, day *P* < 0.01, jet‐lag *P* < 0.05; (D) interaction *P* < 0.05, day *P* < 0.001, jet‐lag *P* < 0.01; (E) interaction *P* < 0.001, jet‐lag ns., phase *P* < 0.01; (F)interaction *P* < 0.0001, jet‐lag ns., phase *P* < 0.0001; (G) interaction *P* < 0.05, jet‐lag *P* < 0.05, phase *P* < 0.05; (H) interaction ns., jet‐lag *P* < 0.05, phase *P* < 0.01.

**Figure 4:**
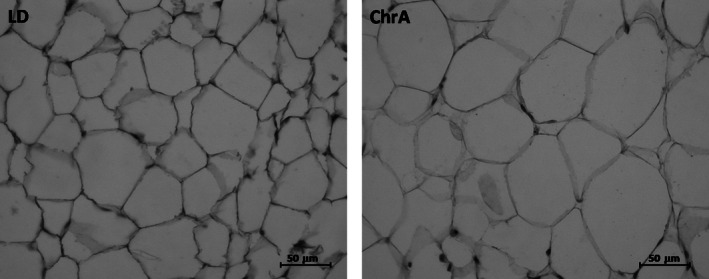
Histological analysis of retroperitoneal adipose tissue indicated an increase in adipocytes size in animals under ChrA (right) as compared to control animals under LD (left). See Figure 3B for quantification and analysis.

We detected several metabolic alterations at different levels in animals under ChrA, some of which varied according to the time spent under the protocol (30 or 60 days after the start of ChrA; JL30 and JL60, respectively). Desynchronized mice showed increases in (mean % of increase vs. LD group): the levels of circulating triglycerides (39%), both retroperitoneal (172%) and epididymal (61%) adipose tissue masses, and in adipocytes area (28%) at JL60 (Fig. 3A–D; see Fig. 4 for histology images of adipose tissue). A slight increase in leptin levels was observed at JL60 (*t‐test, P* < 0.05; mean at JL30 and JL6 ±SD, respectively: ChrA = 2.38 ± 1.21 ng/mL and 5.72 ± 1.52 ng/mL, LD = 2.05 ± 0.77 ng/mL and 4.17 ± 1.76 ng/mL). No differences were found in plasma glucose levels at either time point (mean at JL30 and JL60 ±SD, respectively: ChrA = 1.38 ± 0.30 mg/mL and 1.22 ± 0.21 mg/mL, LD = 1.50 ± 0.38 mg/mL and 1.33 ± 0.29 mg/mL; two‐way ANOVA: interaction, day under ChrA, and jet‐lag all ns.). At the behavioral level, we observed no light–dark variations in neither food nor water intake in mice under the desynchronizing protocol, in contrast to the robust patterns shown by control animals under LD which show maximal values during darkness (Fig. 3E and F). These animals also displayed a suppression of the light/dark patterns of circulating triglycerides and liver weight like those displayed by control mice (Fig. 3G and H), while they maintained light/dark variations in adipocytes area which, as stated above, was increased under ChrA (two‐way ANOVA: interaction ns., light phase *P* < 0.0001, jet‐lag *P* < 0.0001; Bonferroni contrasts to compare light and dark phases values within groups: LD *P* < 0.0001, ChrA *P* < 0.0001).

**Figure 5:**
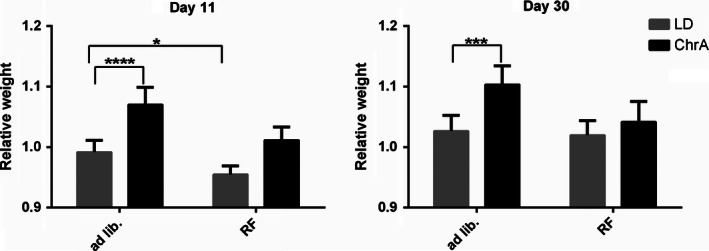
Relative body weight of animals in the restricted feeding experiment, at days 11 and 30 after the start of the protocols (*n* = 6 for all groups). Weights were relativized to those of day 0 when protocols, lighting and/or dark phase‐restricted feeding (RF), started. Data was analyzed through two‐way ANOVA at specific time cuts across the experiment. Dunnett contrasts a posteriori against the control group (ad libitum fed LD group) were also performed (asterisks indicate significant *P* values). Two‐way ANOVA results: Day 11) Weights: interaction ns., feeding *P* < 0,0001, jet‐lag *P* < 0,0001; Day 30) Weights: interaction *P* < 0.05, feeding *P* < 0,01, jet‐lag *P* < 0,001.

To test if the abnormally increased weight gain observed under ChrA could be related to alterations in patterns of food intake, we designed a food restriction experiment in which animals were allowed to feed only during the first 6 h of the dark phases of their light schedule (see Methods). This restricted feeding schedule was phase locked to the ChrA schedule and because of that, to the short‐period component of locomotor activity under forced desynchronization (which is entrained to ChrA; see [Casiraghi et al. 2012]). The experiment then comprised four groups of animals: the ad libitum‐fed (AL) control group under LD, the restricted‐feeding (RF) group under LD, the AL group under ChrA, and the RF group under ChrA. Figure 5 shows the relative weights and daily food intake at 11 days (after the acute effect of the onset of RF) and 30 days after the start of the ChrA and feeding schedules (see Table 1 for absolute weight changes). The RF protocol prevented the abnormal weight gain observed under ChrA at both time points, with no differences in food intake (mean daily intake in grams ± SD: AL + LD = 3.4 ± 0.3, RF + LD = 3.2 ± 0.3, AL + ChrA = 3.3 ± 0.4, RF + ChrA = 3.0 ± 0.3; two‐way ANOVA analysis revealed no differences in intake at any of the time cuts). After 30 days, both RF + LD and RF + ChrA groups displayed weights undistinguishable from the control AL + LD group. The AL + ChrA, as expected, gained significantly more weight throughout the experiment. Interestingly, the RF schedule did not prevent the desynchronization of general locomotor activity rhythms described under ChrA (not shown).

**Figure 6:**
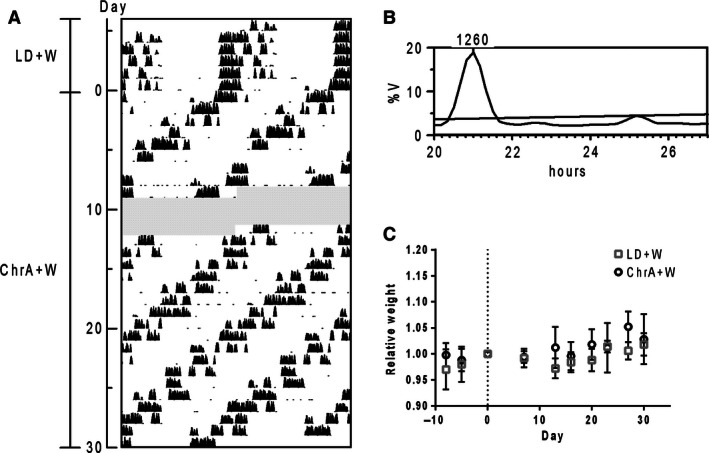
(A) Double‐plot actogram and (B) corresponding Sokolove–Bushell periodogram of wheel running activity of a representative mouse under ChrA with access to a running wheel (ChrA + W) showing correct synchronization to the schedule (grayed areas indicate missing days of recording). The SB periodogram indicates a locomotor activity rhythm with a period of 21 h, consistent with the average period of the schedule (see [Casiraghi et al. 2012]). (C) Relative body weight curves of ChrA + W and control animals (LD + W; *n* = 5 for each group). Day 0 indicates the day of the start of the ChrA schedule, and recorded weights across the experiment are all relativized to the weight on day 0 for each animal. No differences between groups were found after analyzing curves through repeated measures two‐way ANOVA: interaction ns., time *P* < 0.0001, jet‐lag ns.

We then studied whether reinforcing synchronization to ChrA could influence the outcome of the described alterations. We first tested the effect of wheel running on circadian behavior and metabolism under ChrA desynchronizing conditions. The inclusion of a running wheel in the cage of animals under the ChrA protocol completely abolished the desynchronization of locomotor activity rhythms. Animals displayed a single rhythmic component of running activity with a period of 21 h, phase locked to the light schedule (Fig. 6A and B). Access to the running wheel also prevented the observed changes in body weight gain when compared to animals under normal LD conditions which were also allowed to access running wheels, to control for the direct effects of exercise on body weight (Fig. 6C, and Table 1). Daily food intake did not differ between groups (mean daily intake in grams ± SD: ChrA + W = 3.4 ± 0.5, LD + W = 4.0 ± 0.3; repeated measures two‐way ANOVA: ns. for interaction, jet‐lag and day).

We then studied the effect of social interaction on weight gain under ChrA. We observed that housing the animals in groups of 3 or 4 also prevented the alterations in weight gain due to ChrA described for individually caged mice (two‐way ANOVA: interaction *P* < 0.05; time under ChrA *P* < 0.0001; jet‐lag ns.; see Table 1), although because of the experiment constraints, we could not determine neither individual food intake nor activity rhythms under these conditions.

## Discussion

In this paper, we have described the metabolic alterations generated by chronic advancing jet‐lag in mice. The main finding is that animals under this protocol gain (quite early after the start of the protocol) significantly more weight than control animals independently of the amount of food intake. We showed that circulating triglycerides are augmented in desynchronized mice, a factor that has long been described as a marker for further alterations and potentially, metabolic syndrome, among other diseases (Grundy 1998). Moreover, day–night changes of triglycerides in blood were suppressed, similar to what is found in mutants of the clock gene *bmal‐1* (Rudic et al. 2004). We have also detected increased retroperitoneal and epididymal adipose tissue mass, and increased adipocyte size, evident only during the second month under the desynchronization protocol, although fat could have been accumulated before that in other adipose tissues not analyzed in this work. There are evidences that the hyperplasia of adipose tissue per se in “healthy” obese people does not necessarily lead to disease (Ruderman et al. 1998). But, on the other side, reduced adipogenic potential, which drives the increase in adipose mass through the hypertrophy of adipose cells, as we observed in ChrA forced desynchronized mice, is indeed associated with cell dysfunction and metabolic disease (O'Connell et al. 2011). Also, the slight increase in leptin levels detected at JL60 correlates as expected with the increase in adipose tissue mass (Couillard et al. 2000). It is interesting that increased brown adipose tissue, and decreased blood levels of insulin and glucose were observed in rats under a similar advancing CJL schedule, although they did not show abnormal weight gain nor lipid metabolite changes (Herrero et al. 2015).

The observation that abnormal weight gain under ChrA does not correlate with an increase in absolute food intake opens the question about how is this increase mediated. Desynchronized animals could be less active, decreasing basal metabolic rate displaying lower temperatures than controls, so as to explain a higher gain of mass from equal sources of energy. Alterations may in fact be generated by growing fat tissues, although this would be less probable as we found these to increase more acutely during the second month of ChrA. A less probable but still possible alternative could include alterations in the absorption and trafficking of nutrients, which is regulated by circadian elements (Douris et al. 2011). Further experiments should be performed to track down the underlying energetic features of body weight increase under ChrA.

Food intake is controlled in a circadian fashion, and displays a consistent rhythm with the main episodes of feeding during the wake phase. In coincidence, metabolic functions are temporally organized to optimize the use of energy sources. Changing these normal daily phase relationships by restricting food availability to certain phases across the LD cycle, or altering behavioral synchronization to light–dark cycles, has effects on the circadian system and on metabolism. Feeding during the rest phase deeply affects circadian function (Damiola et al. 2000; Salgado‐Delgado et al. 2013), and also induces abnormal weight gain (Arble et al. 2009; Bray et al. 2013). Rest‐phase feeding can alter body weight after only 9 days under this protocol (Bray et al. 2013). Forced desynchronization induced by ChrA appears to be a condition for increased weight gain at least in mice, since animals under this schedule with access to a running wheel, or those under ChrD, protocols that do not induce desynchronization, show no abnormalities. For instance, daily neuroendocrine synchrony of wake state and glucose energy supply has been shown to be of importance, since corticosterone signaling in the liver is impaired at the receptor level by ChrA (Iwamoto et al. 2014). Competing metabolic and circadian interactions can generate such alterations, by transient modulation of retinal input to the SCN, disorganization of SCN neurohumoral outputs, and/or reducing SCN communication with hypothalamic sites responsible for integrating energy homeostasis and circadian signals [reviewed in: (Blum et al. 2012)].

In nocturnal rodents under laboratory conditions, liver weight has been shown to display a rhythmic pattern with maximum values during the day, 12 h after the main nocturnal feeding episodes, and forcing animals to feed at different times has a direct effect on the pattern of liver weight (Sitren and Stevenson 1978; Stokkan et al. 2001). We have shown that there are no light–dark differences in the amount of food intake under ChrA, and moreover, that normal daily variations in liver weight are abolished. Nevertheless, our experiments cannot rule out defined rhythms of feeding behavior in desynchronized animals. An experiment measuring feeding and/or drinking in animals with precision across several complete ChrA cycles (and taking both desynchronized components of locomotor activity into account) should be carried out to completely characterize such behaviors in desynchronized animals.

The schedule to force mice under ChrA to feed exclusively at the dark phases was designed so to phase lock the availability of food to the light–dark schedule, and hence to the synchronized locomotor activity component (Casiraghi et al. 2012). Restricting food availability to the first 6 h of each dark phase managed to rescue the abnormal increase of body weight under ChrA across 30 days of the protocol, without modifying the levels of intake. However, it did not abolish desynchronization of locomotor activity rhythms, indicating that the rapid initial weight gain under ChrA may be due to a desynchronization between locomotor rhythms, the pattern of food intake (or the lack of such pattern), and rhythms in metabolic function. Restricting food to the night was shown to help overcome metabolic (Salgado‐Delgado et al. 2010) and even immune alterations (Guerrero‐Vargas et al. 2015) in other models of internal desynchronization, such as that of rest‐phase forced activity in rats (in which patterns of feeding are also disrupted). The restricted feeding experiment was designed taking into account two possible effects of the intervention. Food availability (1) could work as reinforcement for the light‐dark schedule, allowing animals to cope with the desynchronizing protocol; and/or (2) that it would be coincident with optimal metabolic rhythms under ChrA (that may be in turn induced by the feeding schedule per se). Regarding the first possibility, while food availability has neither effect on activity rhythms driven by the SCN (Hara et al. 2001), nor on its entrainment to desynchronizing 22‐h light–dark cycles in rats (Angles‐Pujolras et al. 2006), it can entrain several peripheral clocks related to feeding and nutrient processing (Schibler et al. 2003). In our model, restricted feeding could induce changes in peripheral clocks and metabolic function so that the second possibility could be met to overcome the alterations in body weight.

Wheel running has been shown to function as a feedback signal for the circadian clock, helping animals synchronize to shortened light–dark cycles (Chiesa et al. 2005). We found that volitional access to running wheel under ChrA, both prevented the forced desynchronization of locomotor activity rhythms and was accompanied by normal body weight gain, as compared to control animals under LD also allowed to access a running wheel. No alterations of body weight were either detected under the ChrD protocol, a CJL schedule symmetrical to ChrA consisting in chronic delays of the light–dark cycle which does not produce desynchronization of locomotor rhythms (Casiraghi et al. 2012).

We found that housing mice in groups was able to prevent the alterations in weight gain under ChrA, a fact that suggests a role for social contact in adaptation to circadian disrupting conditions. Social interaction is an excellent example of a well‐characterized nonphotic class of stimulus, as well as an important feedback element within the circadian system (Mistlberger and Skene 2004). Group contact can modulate biological rhythms in hamsters (Mrosovsky 1988), degus (Goel and Lee 1995), squirrels (Rajaratnam and Redman 1999), rats (Cambras et al. 2011), and mice (Crowley and Bovet 1980; Viswanathan 1999). There is a chance, then, that social interaction might reinforce synchronization to the ChrA schedule through nonphotic feedback, helping overcome metabolic disturbances.

Animal models of circadian disruption also display a vast array of metabolic alterations. Either genetic disruption of the circadian clock (Turek et al. 2005), forcing nocturnal animals to be active during the day (Salgado‐Delgado et al. 2008) or the manipulation of the light–dark cycle period (Karatsoreos et al. 2011; Herrero et al. 2015) can alter metabolic function in diverse ways. Among the most commonly reported health complications suffered by shift‐ and night‐workers are those related to metabolism, mainly obesity but also others contributing to metabolic syndrome (Golombek et al. 2013). These disorders are not only deleterious per se, but also because of the potential deregulation of other systems and the higher risk for other diseases, such as cancer (Sahar and Sassone‐Corsi 2009). In addition, three factors arise from our results that should be analyzed in the evaluation of therapeutic strategies to improve health and quality of life in human shift‐workers: stimuli that can reinforce synchronization (exercise and social contact), adequate temporization of feeding regimes, and optimization of the design of working shifts (delaying schedules vs. advancing schedules).

This work has provided evidence on the consequences of circadian misalignment between metabolic and behavioral rhythms, which promote increments in adipose tissue mass, changes in adipocyte size, alterations in rhythms of blood triglycerides, and even globally affecting body weight of the animals. Forcing the desynchronized animals to feed on restricted schedules normalizes body weight gain. This was observed also when promoting circadian synchronization either by delaying CJL schedule, or by volitional exercise on running wheels under advancing CJL. Both manipulations suggest that either central and/or peripheral synchronization, are key components for metabolic and behavioral interactions sustaining weight homeostasis. Further studies must be pursued to determine the specific mechanisms by which circadian desynchronization leads to metabolic alterations, and potentially metabolic syndrome and obesity.

## Conflicts of Interest

None declared.
